# Work Function Adjustment by Using Dipole Engineering for TaN-Al_2_O_3_-Si_3_N_4_-HfSiO_x_-Silicon Nonvolatile Memory

**DOI:** 10.3390/ma8085112

**Published:** 2015-08-07

**Authors:** Yu-Hsien Lin, Yi-Yun Yang

**Affiliations:** Department of Electronic Engineering, National United University, No. 1, Lienda, Miaoli 36003, Taiwan; E-Mail: yun70076@gmail.com

**Keywords:** TaN-Al_2_O_3_-HfSiOx-SiO_2_-Silicon (TAHOS), nonvolatile memory (NVM), dipole engineering, work function

## Abstract

This paper presents a novel TaN-Al_2_O_3_-HfSiO_x_-SiO_2_-silicon (TAHOS) nonvolatile memory (NVM) design with dipole engineering at the HfSiO_x_/SiO_2_ interface. The threshold voltage shift achieved by using dipole engineering could enable work function adjustment for NVM devices. The dipole layer at the tunnel oxide–charge storage layer interface increases the programming speed and provides satisfactory retention. This NVM device has a high program/erase (P/E) speed; a 2-V memory window can be achieved by applying 16 V for 10 μs. Regarding high-temperature retention characteristics, 62% of the initial memory window was maintained after 10^3^ P/E-cycle stress in a 10-year simulation. This paper discusses the performance improvement enabled by using dipole layer engineering in the TAHOS NVM.

## 1. Introduction

Nonvolatile memory (NVM), because of its high density and low cost, is widely used for portable mass storage purposes in digital cameras, tablet PCs, and smartphones [1]. A crucial challenge in the electronics industry is obtaining low-power fast NVM devices with small dimensions. A silicon-oxide-nitride-oxide-silicon (SONOS)-like structure has become widely used for charging devices because it does not have a planar scaling problem for floating gate isolation and exhibits considerable potential for achieving high program/erase (P/E) speeds, low programming voltages, and low power performance [1,2,3,4,5,6,7].

Silicon-Oxide-Nitride-Oxide-Silicon (SONOS) flash memory devices are potential candidates for replacing conventional floating-gate NAND (Not AND) flash devices in the sub-32 nm technology node [6,7]. SONOS-like devices have several advantages over the conventional floating-gate device, such as rapid programming, low-power operation, high-density integration, and excellent reliability.

According to studies on SONOS flash, TaN-Al_2_O_3_-Si_3_N_4_-SiO_2_-silicon (TANOS) structure flash memory [8,9,10] exhibits excellent performance because of its immunity to gate injection when metal gate TaN with a high work function is used. Moreover, several studies have presented various types of high-*k* dielectric trapping layers as potential candidates for replacing Si_3_N_4_ to provide discrete NVM charge storage [11,12,13,14,15,16]. Furthermore, high-*k* dielectric materials can improve the gate capacitance, and maintain an equivalent potential difference for a greater thickness compared with SiO_2_. Therefore, the leakage through the dielectric can be minimized, and the scaling limits can be extended. Moreover, to achieve a large memory window for differentiating between stable programs and erased states, a high-*k* dielectric trapping layer can provide sufficiently high trapping density for charge storage [17]. According to the International Technology Roadmap for Semiconductors, high-*k* trapping layer use in flash memory has high potential for scalability below the 32-nm node [18].

Metal gate electrodes with high-*k* dielectric oxide may be made more effective than poly-Si by improving the carrier mobility, thus avoiding the poly-Si depletion effect and dopant penetration through the gate oxide [19,20,21,22]. However, using a metal gate layer requires *n*- and *p*-type metals with appropriate work functions for targeting the suitable threshold voltage (V_th_) for high-performance complementary metal-oxide-semiconductor (CMOS) logic applications on bulk Si [23,24]. In addition, studies have demonstrated V_th_ shift caused by dipole formation at high-*k*/SiO_2_ interfaces [25,26,27]. The areal density difference of oxygen atoms is the driving force in dipole formation at these interfaces [28,29]. In this study, we used the dipole engineering for NVM to modulate V_th_ with different high-*k* dielectric layers. The proposed design with adjustable V_th_ exhibited excellent characteristics such as a considerably large memory window, high-speed P/E, excellent endurance, and optimal disturbance.

## 2. Experimental Section

**Figure 1:**
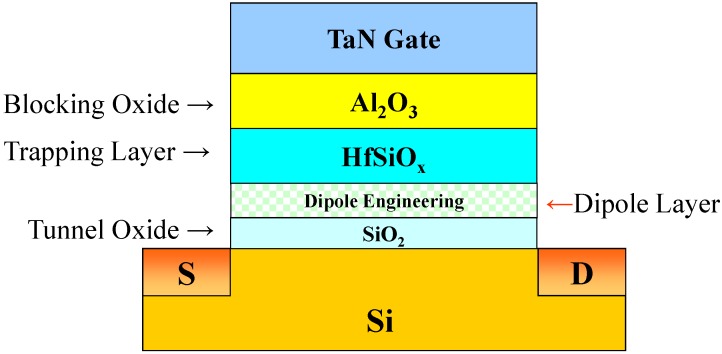
Cross-sectional cell structure of the TAHOS NVM device using dipole engineering.

Figure 1 illustrates the structure of our TAHOS SONOS-like NVMs. These devices were fabricated on 6 inch Si wafers. After the active region was patterned, a 4 nm oxide tunnel was thermally grown at 1000 °C in a vertical furnace system. Next, 1 nm HfO_2_ and Al_2_O_3_ thin films, used in the dipole layer, were deposited using metal–organic chemical vapor deposition (MOCVD). We compared three samples: one without a dipole layer, one with a 1 nm HfO_2_ dipole layer, and one with a 1 nm Al_2_O_3_ dipole layer. Table 1 compares the devices. After a 10 nm trapping HfSiO_x_ deposition, MOCVD was used to deposit a 10 nm Al_2_O_3_ thin film, which was used as a blocking oxide. Next, a 100 nm TaN layer was deposited using a sputtering method. After gate patterning, a self-aligned implantation was used to create an n^+^ source/drain with As^+^ at a dose of 5 × 10^15^ cm^−2^ and energy of 15 keV. Dopant activation and the interaction of the dipole layer with tunnel oxide were accomplished through rapid thermal annealing (RTA) at 950 °C for 15 s. The remainder of the subsequent standard CMOS procedures were completed for fabricating the TAHOS SONOS-like NVM devices.

**Table 1:** Dipole engineering for TAHOS NVM devices.

Dipole engineering	w/o Dipole	w/i Dipole Al_2_O_3_	w/i Dipole HfO_2_
**Tunneling oxide**	SiO_2_ 40 Å	SiO_2_ 40 Å	SiO_2_ 40 Å
**Dipole layer**	–	Al_2_O_3_ 10 Å	HfO_2_ 10 Å
**Trapping layer**	HfSiO_x_ 100 Å	HfSiO_x_ 100 Å	HfSiO_x_ 100 Å
**Blocking oxide**	Al_2_O_3_ 100 Å	Al_2_O_3_ 100 Å	Al_2_O_3_ 100 Å

## 3. Results and Discussion

**Figure 2:**
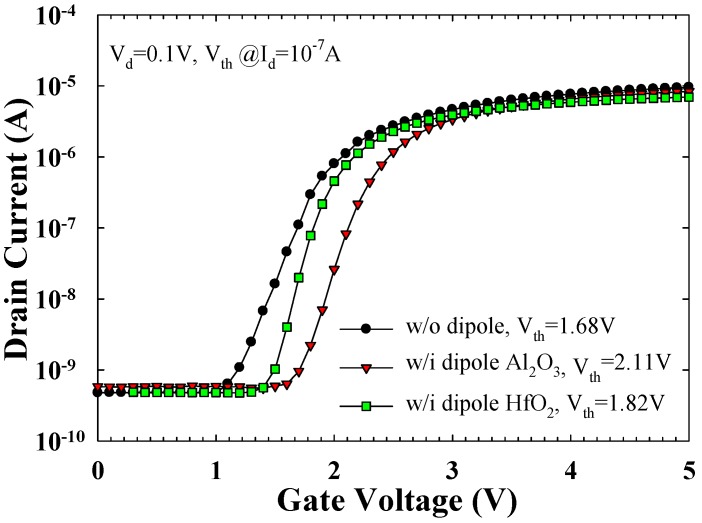
I_d_–V_g_ curve of the TAHOS NVM devices.

Figure 2 plots the I_d_-V_g_ curve of the proposed TAHOS NVM devices. The drain voltage (V_d_) of the I_d_-V_g_ curve is 0.1 V, and V_g_ transverses from 0 to 5 V. The V_th_ at 10^−7^ A I_d_ is 1.68, 2.11, and 1.82 V for the without dipole, Al_2_O_3_ dipole, and HfO_2_ dipole samples, respectively. Al_2_O_3_/HfO_2_ dipole layer incorporation in the TAHOS stacks results in a positive V_th_ shift in the NVM devices. The V_th_ tuning was found to be proportional to the net dipole moment associated with the Hf-O/Si-O and Al-O/Si-O bonds at the high-*k*/SiO_2_ interface because of electronegativity and areal density difference of oxygen atoms [28,29]. According to the electrical measurement results, the dipole effects caused by the interfacial Al_2_O_3_ and HfO_2_ dipole layer shift the effective work function toward p-metal. Therefore, different dipole layers can be used for V_th_ adjustment for tuning the conventional gate electrode work function.

**Figure 3:**
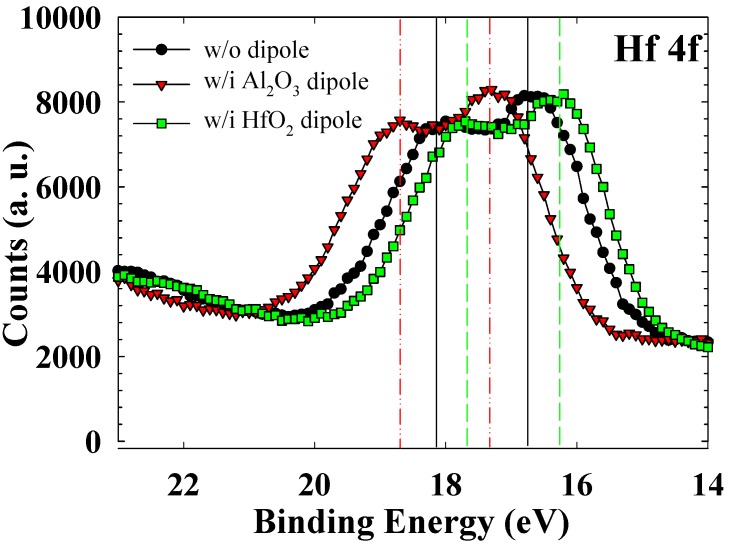
Hf 4f XPS spectra of the samples without dipole, with Al_2_O_3_ dipole, and with HfO_2_ dipole.

X-ray photoelectron spectroscopy (XPS) was performed by using an Al Kα X-ray source (1486.6-eV photons) to determine the bonding environments of the Hf atoms. Figure 3 shows the Hf 4f photoemission peaks of the samples without dipole, with Al_2_O_3_ dipole, and with HfO_2_ dipole. The test sample for XPS was prepared for the without dipole or with Al_2_O_3_ dipole layer following preparation of the HfSiO_x_ thin film after RTA at 950 °C for 15 s. In the without dipole sample, we observed well-defined 4f_5_*_/_*_2_ and 4f_7__/__2_ feature peaks for the HfSiO_x_ thin film that correspond to Hf–O–Si bonding. For the HfO_2_ dipole sample, these peaks shifted to lower binding energies (4f_5_*_/_*_2_: *ca.* 17.7 eV; 4f_7_*_/_*_2_: *ca.* 16.2 eV), resulting in HfO_2_ dipole formation after RTA [30]. Moreover, for the Al_2_O_3_ dipole sample, these peaks shifted to higher binding energies (4f_5_*_/_*_2_: *ca.* 18.7 eV; 4f_7_*_/_*_2_: *ca.* 17.2 eV), resulting in Al_2_O_3_ dipole formation after RTA [31]. The XPS results provide definite evidence of HfO_2_ and Al_2_O_3_ dipole formation through dipole engineering.

**Figure 4:**
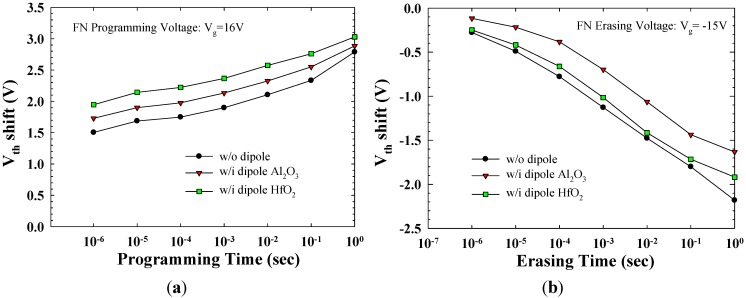
(**a**) Program characteristics of the TAHOS NVM devices; (**b**) Erase characteristics of the TAHOS NVM devices.

Figure 4a,b presents the P/E characteristics of various pulse widths for different operation conditions. The P/E operations were performed using Fowler–Nordheim tunneling at V_g_ = 16 V and V_g_ = −15 V with V_d_ = V_s_ = 0 V. The V_th_ shift is defined as the threshold voltage change of a device between the written and the erased states. ΔV_th_ increased with the P/E pulse time and bias, and the memory window was >1.5 V. In conventional flash memory, >0.8 V memory window is sufficient for judge the “1” or “0” state, the V_th_ window of the device between program/erase state is enough for flash memory operation. No erase saturation effect occurred even at high erase bias or over a long erase time because of TaN’s high work function (4.7 eV), which prevents the injection of electrons from the gate [10]. Regarding the dipole splits, the Al_2_O_3_ and HfO_2_ dipole samples have higher programming speeds than does the without dipole sample because of the low barrier height for electron tunneling. The Al_2_O_3_ or HfO_2_ dipole samples have slightly lower erasing speeds because of the increasing thickness of gate oxide.

**Figure 5:**
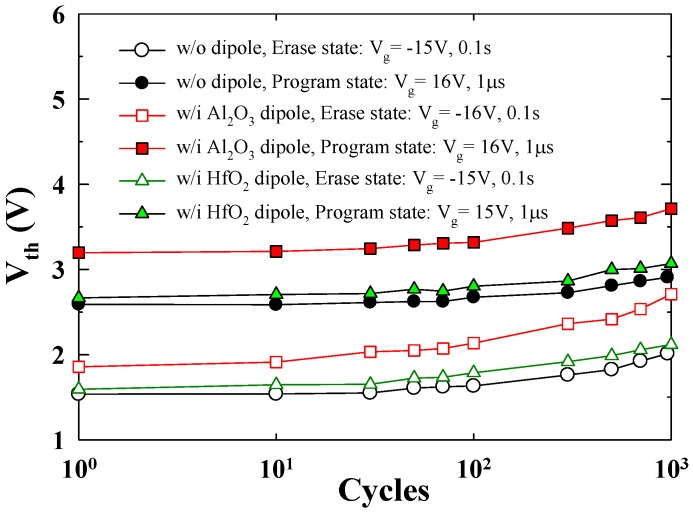
Endurance characteristics of the TAHOS NVM devices.

Figure 5 plots the endurance characteristic of the proposed TAHOS NVM devices. To achieve approximately the same memory window, we used the following P/E conditions: V_g_ = 16 V, 1 us/V_g_ = −15 V, and 0.1 s for the without dipole sample; V_g_ = 16 V, 1 us/V_g_ = 16 V, and 0.1 s for the Al_2_O_3_ dipole sample; and V_g_ = 16 V, 1 us/V_g_ = −16 V, and 0.1 s for the HfO_2_ dipole sample. The NVM device displayed more favorable endurance, retaining 75% of its initial memory window after 10^3^ P/E cycles. For the endurance characteristics, higher erasing V_t_ after cycling is the reliability issue in the conventional flash memory for thick tunnel oxide degradation [2,6,8]. This result is because the degradation of the tunnel oxide (SiO_2_) in TAHOS NVM devices mainly depends on the electrical field. In addition, the endurance curves increase slightly as the number of P/E cycles increase, because of the formation of operation-induced trapped electrons. This is intimately related to the use of thick tunnel oxide and presence of minute residual charges in the SiO_2_ after cycling.

**Figure 6:**
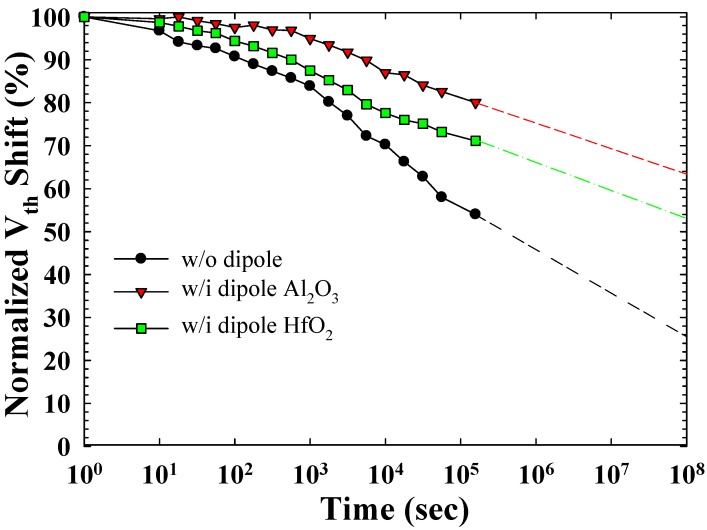
Retention characteristics of the TAHOS NVM devices.

Figure 6 illustrates the retention characteristics with 10^3^ P/E cycled stress condition of the proposed TAHOS NVM at a high temperature (T = 85 °C). The retention time was up to 10^8^ s for 38%, 48%, and 72% charge losses for the Al_2_O_3_ dipole, HfO_2_ dipole, and without dipole samples, respectively. The retention of both of the dipole samples was superior to that of the without dipole sample because of the formation of a thick tunnel oxide. Moreover, the Al_2_O_3_ dipole sample exhibited superior retention to that of the HfO_2_ dipole sample because the Al_2_O_3_ layer has a greater electron barrier height [31].

## 4. Conclusions

TAHOS NVM was fabricated using an Al_2_O_3_/HfO_2_ dipole layer at an HfSiO_x_/SiO_2_ interface, demonstrating a V_th_ shift and providing work function adjustment. A 2-V memory window was achieved by applying 15 V for only 10 μs. Regarding endurance, a 1-V of memory window was maintained after 10^3^ P/E stress cycles. Regarding retention, 62% of the initial memory window was maintained after a 10-year simulation at high temperature (T = 85 °C). Thus, dipole engineering has great potential for work function adjustment in conventional SONOS-type NVM.
